# Self-renewal potential of NPCs decreased in vitro during human embryonic brain development with reduced activation of mitogen-activated protein kinases signaling

**DOI:** 10.1186/1750-1326-7-S1-S19

**Published:** 2012-02-07

**Authors:** Lingyu Zhao, Qian Jiao, Xinlin Chen, Pengbo Yang, Bingqiao Zhao, Ping Zheng, Yong Liu

**Affiliations:** 1 Institute of Neurobiology, Xi'an Jiaotong University College of Medicine, 710061, China; 2 The State Key Laboratory of Medical Neurobiology, Shanghai, 200032, China

## Background

Neural progenitor cells (NPCs) are multipotent and self-renewing cells during development and throughout adulthood. A key question in NPCs transplantation therapies is which stage NPCs from human embryonic brain development are ideal donor.

## Methods

In this study, we investigated the difference of survival, proliferation and apoptosis of NPCs from 12 w, 16 w and 20 w human embryonic brain, and the phosphorylation of mitogen-activated protein kinases (MAPKs) signaling molecules. The NPCs survival was evaluated by trypan blue staining. The NPCs proliferation was evaluated by 3-(4,5-dimethylthiazol-2-yl)-2,5-diphenyltetrazolium bromide (MTT) assay, diameter measurement of neurospheres and cell cycle analysis. The cell death of NSCs was evaluated by Hoechst staining. The expression of phosphorylated extracellular signal regulated kinase (ERK), c-Jun N-terminal protein kinase (JNK) and p38 were analyzed by immunoblotting assay.

## Results

The results showed that the survival of human NPCs gradually decreased with human embryonic brain development in vitro; the NPCs proliferation gradually decreased in cell activity, diameter of neurospheres and cell division with human embryonic brain development, and the NPCs apoptosis gradually increased. Phosphorylation of ERK1/2 gradually decreased with human embryonic brain development, however phosphorylation of p38 MAPK gradually increased, and there were no significant change in p-JNK2 level.

## Conclusions

The results suggest that self-renewal potential of NPCs decreased in vitro during human embryonic brain development, the activation of ERK signaling pathway were decreased in the process, and human NPCs from 12-week fetuses cortex were ideal donor for cell transplantation therapy because of their advantage in terms of survival and proliferation at this period.

